# Exploring Eye Movement Biometrics in Real-World Activities: A Case Study of Wayfinding

**DOI:** 10.3390/s22082949

**Published:** 2022-04-12

**Authors:** Hua Liao, Wendi Zhao, Changbo Zhang, Weihua Dong

**Affiliations:** 1 School of Geographic Sciences, Hunan Normal University, Changsha 410081, China; liaohua@hunnu.edu.cn (H.L.); zhaowendi@hunnu.edu.cn (W.Z.); cat_zcb@hunnu.edu.cn (C.Z.); 2 Hunan Key Laboratory of Geospatial Big Data Mining and Application, Hunan Normal University, Changsha 410081, China; 3 State Key Laboratory of Remote Sensing Science, Beijing Key Laboratory for Remote Sensing of Environment and Digital Cities, Faculty of Geographical Science, Beijing Normal University, Beijing 100875, China

**Keywords:** mobile and wearable eye tracking, stimulus-independent biometric recognition, biometric identification and verification, pedestrian navigation, fixation semantic features

## Abstract

Eye movement biometrics can enable continuous verification for highly secure environments such as financial transactions and defense establishments, as well as a more personalized and tailored experience in gaze-based human–computer interactions. However, there are numerous challenges to recognizing people in real environments using eye movements, such as implicity and stimulus independence. In the instance of wayfinding, this research intends to investigate implicit and stimulus-independent eye movement biometrics in real-world situations. We collected 39 subjects’ eye movement data from real-world wayfinding experiments and derived five sets of eye movement features (the basic statistical, pupillary response, fixation density, fixation semantic and saccade encoding features). We adopted a random forest and performed biometric recognition for both identification and verification scenarios. The best accuracy we obtained in the identification scenario was 78% (equal error rate, EER = 6.3%) with the 10-fold classification and 64% (EER = 12.1%) with the leave-one-route-out classification. The best accuracy we achieved in the verification scenario was 89% (EER = 9.1%). Additionally, we tested performance across the 5 feature sets and 20 time window sizes. The results showed that the verification accuracy was insensitive to the increase in the time window size. These findings are the first indication of the viability of performing implicit and stimulus-independent biometric recognition in real-world settings using wearable eye tracking.

## 1. Introduction

Every person is unique. The uniqueness of a person is associated with his or her biological (e.g., face, fingerprint, iris and DNA) and behavioral (e.g., handwriting, voice and eye movements) traits. These unique biological or/and behavioral traits can be used to recognize a person, which is referred to as *biometric recognition*, or simply *biometrics* [1]. Currently, the fingerprint, face and iris are the three most commonly used traits and have many practical applications in forensics, surveillance and everyday life (e.g., unlocking smartphones and laptops) due to their distinct advantages over passwords and tokens [2].

Using eye movements to recognize persons was pioneered by Kasprowski and Ober [3] and has gained attention since then [4]. One advantage of eye movement biometrics is that eye movements cannot be forged. As Holland and Komogortsev noted, “eye movements are uniquely counterfeit resistant due to the complex neurological interactions and the extraocular muscle properties involved in their generation” (p. 1) [5]. Another advantage is that eye movements can provide continuous biometric recognition in an unobstructive way [6,7]. More importantly, with the development of eye tracking technology, mobile and wearable eye trackers are becoming lighter, cheaper and more accurate and thus may become ubiquitous sensors in the near future [7]. Thus, mobile and wearable eye movement biometrics (MWEMB) may be performed in unconstrained environments and in real-world activities. Furthermore, in real-world applications, MWEMB can be implicitly performed and easily combined with other gaze-based techniques, such as human activity recognition [8] and attentive user interfaces [9], to provide a more personalized and tailored experience in human–computer interactions (HCIs) [10]. To date, although mobile and wearable eye tracking has been widely used in real environments to investigate human gaze behavior (e.g., [11,12,13,14,15,16]), eye movement biometric studies have only been conducted in controlled laboratories.

Aside from the technical issues of eye movement biometrics in wearable devices [17,18], there are multiple challenges to performing MWEMB in real-world applications in real environments. In this study, we specifically focus on two challenges: implicity and stimulus independence.

- *Implicity* refers to recognizing persons when they have no awareness of being recognized, or recognizing persons without interfering with their actions at hand. Implicity is important for continuous recognition because asking users to perform explicit recognition constantly is annoying, or users are involved in highly concentrated activities (e.g., banking transactions) that cannot be disturbed.
- *Stimulus independence* refers to recognizing persons using stimuli that have not been trained by a classifier (i.e., have not been seen by the persons) [7,19]. For example, a stimulus-independent biometric system can recognize persons when they are watching natural images, but the system was trained using face images. In the stimulus-independent scenario, the training and testing sample are not matched, which can interfere with the learning effects of users.

Implicity and stimulus independence are two closely related properties of an eye movement biometric system in a real-world context and are difficult to realize in machine learning [19]. Imagine a biometric system that can recognize persons implicitly in everyday activities, such as browsing web pages in the office or wayfinding in a new place, using methods other than fixating on a static cross or following a “jumping” point on a computer screen.

Taking wayfinding in real environments as a case, this study aims to explore implicit and stimulus-independent eye movement biometrics in real-world activities. We used real environments as stimuli and collected subjects’ eye movement data as they were wayfinding in a real environment. The wayfinding task was deemed appropriate for the present study because wayfinding in familiar and unfamiliar environments is a frequently performed activity in everyday life. Wayfinding involves multiple spatial cognitive processes, such as spatial orientation, self-localization and spatial knowledge acquisition, which have been studied extensively in psychology and geography [12,20,21].

To the best of our knowledge, this is the first study exploring the use of wearable eye movement biometrics in real-world activities. Our contributions are as follows.

- We provide the first empirical evidence of the feasibility of implicit and stimulus-independent biometric identification and verification via wearable eye tracking in real environments.
- We compared the performance of five feature sets of eye movements to understand their ability to recognize individuals in real environments. We also tested 20 time windows to determine how much time was sufficient for eye movement biometrics in real-world activities.

In the next section, we briefly introduce eye movement biometric studies (Section 2.1). We then present potential applications of MWEMB and summarize the difficulties of conducting MWEMB in real-world activities (Section 2.2). In Section 3, we detail the wayfinding experiment and biometric recognition methods. We present the results of both the identification and verification scenarios in Section 4. The results are discussed in Section 5, and conclusions are presented in Section 6.

## 2. Background and Related Work

### 2.1. Eye Movement Biometrics in Laboratory

Kasprowski and Ober [3] first explored eye movements in biometrics. They used “jumping” points as stimuli and a Cepstrum transform for the features. On a nine-subject dataset, the K-Nearest Neighbors (KNN, K = 3) performed best, with an average false acceptance rate of 1.48% and an average false rejection rate of 22.59%. “Jumping” point stimuli have been used in many other studies (e.g., [22,23,24]). Another similar stimulus is a moving or static cross, which was used in [25].

Subsequent studies adopted more complex stimuli, such as text, face images and natural images, for eye movement biometrics. For instance, Holland and Komogortsev [23] used text as stimuli and extracted 14 features (called “complex eye movement patterns”), such as fixation count, mean fixation duration and scanpath length. The best equal error rate (EER) achieved was 28% using the text stimuli. Rigas et al. [26] presented a graph matching technique to represent fixations that were collected from subjects’ observing face images. The best accuracy was 70.2% using KNN (K = 3). Cantoni et al. [27] used face images as stimuli and proposed a graph-based method to represent fixation points. They applied the method to 112 subjects, and the best EER they achieved was 22.4%. Saeed [6] explored using eye movements during scene understanding for biometric identification, and the best identification rate reached was 85.72%.

Studies have also explored using video stimuli. For example, Kinnunen et al. [7] presented a 25-min movie as stimuli and recorded 17 subjects’ eye movement data. The data were divided into segments with different durations to explore the impact of duration on biometric identification. They used a Gaussian mixture model to extract eye movement features, and the best EER they achieved was 29.4%. Rigas et al. [28] also used movie stimuli and had a pool of 100 subjects. They segmented the recordings into 2-s intervals and constructed fixation density maps for each interval to use as the features. Their best identification rate was 35.5%.

Schröder et al. [19] tested the robustness of eye movement biometrics using a stimulus-independent classification. They trained a classifier using the TEX dataset (subjects read text) and tested the classifier using the RAN dataset (subjects follow a random dot), and vice versa. They proposed a ‘RDF’ method and achieved a best accuracy of 23.5% for TEX->RAN and 7.8% for RAN->TEX. They also compared the performance by varying trajectory length and found that 90 s of eye movement data could achieve 86.7% accuracy.

More recently, Liao et al. [29] conducted eye movement identification using geo-spatial tasks. They recorded 32 subjects’ eye movement data when they were viewing 40 images that contained a street view and cartographic maps. For each image, the subjects were required to find visual cues from the image to determine where they were and which direction they were facing (i.e., 40 tasks). They used a leave-one-task-out cross-validate approach to test the stimulus-independent performance. By combining a large set of eye movement features and training a random forest classifier, they achieved a best accuracy of 89% with 2.7% EER.

In summary, the performance of eye movement biometrics has improved significantly since it was first proposed in 2004 [3]. Refer to [4,30] for detailed reviews. However, the current studies were conducted in a controlled laboratory where head and body movements were limited.

### 2.2. Eye Movement Biometrics in Real Environment

As mentioned in the Introduction, eye trackers are becoming more portable and inexpensive. Eye tracking is no longer limited to scientific researchers; it is also available to the general population. Eye tracking has been used in personal computers (e.g., Alienware m17 R2 Gaming Laptop [31]), head-mounted displays (e.g., HTC VIVE Pro Eye [32] and Microsoft HoloLens 2 [33]), smartphones (e.g., Huawei Mate 30 Pro) and driver monitoring systems (e.g., BMW [34]) [10,29,35]. The widespread usage of eye tracking expands the possibilities of performing MWEMB in real-world activities, which is critical in the following two scenarios.

First, MWEMB can support continuous verification in an unobstructive way, which can help to prevent ‘hijacking’ attacks [17,36]. The ‘hijacking’ attacks occur when the attacker deprives the access to a system from an authenticated user (i.e., the user has logged-in the system). Therefore, continuous verification is important for highly secure environments such as banking transactions, aircraft cockpits, and defense establishments [36].

Second, MWEMB can be easily combined with other sources of data (e.g., history data) to offer a more personalized and tailored experience in gaze-based HCIs. Consider the following scenario: a wayfinder is walking to a crossroad and needs to check his/her navigation assistant for turning direction. By simply looking at the navigation assistant on his/her smartphone, the user is verified and logged in automatically. Based on the user’s position and direction, the assistant then provides turning information. Furthermore, when the user searches for points of interest, the assistant can make personalized recommendations based on the user’s history data (e.g., previous activities).

However, performing MWEMB in real-world settings faces more challenges than in the laboratory. It is difficult to achieve high experimental control in real environments. Difficulties include [37]:

- **Dynamic visual stimuli**. The real environment is dynamic, and subjects are unconstrained, meaning that different subjects are presented with different visual stimuli (although the static objects of the environment remain stable, such as the terrain, buildings and trees). The dynamic environment cannot be controlled. As a result, eye movement data from different subjects or groups are difficult to compare directly.
- **Subject organization**. It takes time to move subjects from one location to another. In the real world, walking a long distance can add to subjects’ physical stress. Furthermore, subjects may become familiar with the new surroundings while adjusting to them, introducing bias to the findings if familiarity is an influencing factor in the study.
- **Data quality**: The quality of eye tracking data can be easily affected by light condition changes, moving objects, and large head and body movements in the environment. Furthermore, typical mobile eye trackers (e.g., Tobii Pro Glasses: 50~100 Hz [38] and SMI eye tracking glasses: 60 Hz [39]) have a lower tracking frequency than laboratory-based eye trackers (e.g., Tobii Pro Spectrum: up to 1200 Hz [40] and EyeLink 1000 Plus: up to 2000 Hz [41]). Holland and Komogortsev [23] recommended using eye tracking at a frequency greater than 250 Hz for reliable biometric recognition. As a result, low-frequency mobile eye trackers may be unable to differentiate micro-characteristics in subjects’ saccades.

Some of these difficulties cannot be resolved. Researchers have to compromise between maximum experimental control and the ecological validity of the MWEMB. However, evidence of eye movement biometrics in real environments is rare. We used urban wayfinding as a real-world scenario in this study to investigate the possibility of implementing implicit and stimulus-independent MWEMB.

## 3. Methods

### 3.1. Data Collection

Forty-four subjects’ (20 female and 24 male, age: 18–29, M = 23.0, and SD = 2.5) eye movement data were collected from two real-world wayfinding experiments that were previously reported in [12] (Experiment 1) and [16] (Experiment 2) for completely different purposes. The subjects were university students from various backgrounds (e.g., geography, psychology, engineering, arts and management). All subjects had normal or corrected-to-normal vision. They were unaware of the purpose of this study, but they agreed that their data can be anonymously analyzed for scientific research. They were compensated for their participation.

**Figure 1:**
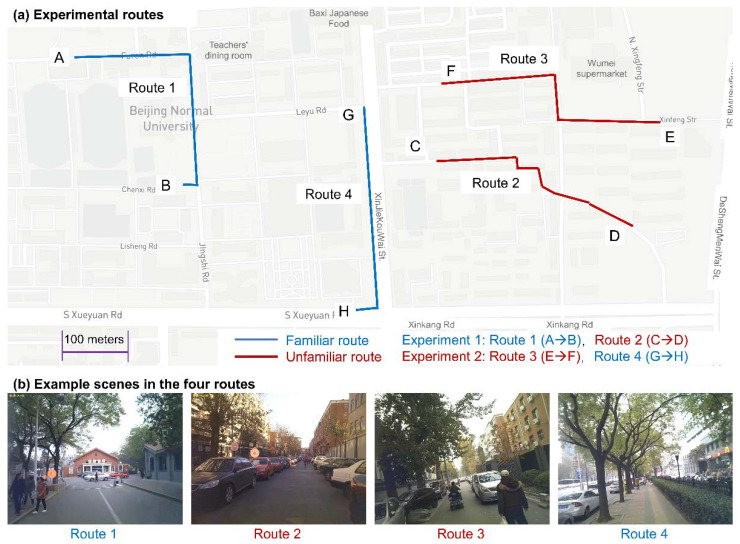
Experimental routes and example scenes. (**a**) Experimental routes and (**b**) example scenes of the real-world wayfinding experiments.

The experiments were conducted on sunny or partly cloudy days. The experimental areas were in Beijing, China. In the experiments, the subjects were required to complete route-following tasks on four routes (Routes 1~4) (Figure 1a). Routes 1 and 4 were located within the university campus with which the subjects were familiar, whereas Routes 2 and 3 were located in a residential area with which the subjects were unfamiliar. In each route, the subjects were asked to follow a predefined path and walk from the start to the end of the path. The subjects were given a printed A4 map with the predefined path highlighted on it so they could look at the map whenever needed. Each route was approximately 500 m long. There was no time limit to complete the tasks. The subjects were also required to complete additional tasks (e.g., searching for targets on the map, freely viewing the map and the environment, and memorizing the routes and filling out questionnaires), but these tasks belonged to another project and were not analyzed in this study. Example scenes from the four routes are shown in Figure 1b. Each subject took approximately 90 min to finish the experiments. Each subject on each route produced a recording, resulting in 176 (44 × 4) recordings in total.

Although the data were collected from the two experiments, the subjects and the protocols (i.e., apparatus and procedure) of the two experiments were identical. The two experiments were used to relieve the subjects’ fatigue because they needed to concentrate on wayfinding tasks and walk long distances in real-world environments. Note that both experiments contained a familiar and unfamiliar route. This was because wayfinding is widespread in both familiar and unfamiliar situations. The subjects’ familiarity level was self-reported by a questionnaire (7-point scale, from 1: very unfamiliar to 7: very familiar). However, we will not distinguish data from familiar and unfamiliar environments in the following analysis because it was outside of the scope of the study.

We used SensoMotoric Instruments (SMI) eye tracking glasses (ETG, Apple, the United States, https://www.apple.com, accessed on 6 April 2022) to collect subjects’ eye movement data (60 Hz, binocular), pupillary response data (pupil diameter) and synchronized forward scene video data (24 fps, 1280 × 960 pixels). The ETG was connected to a Thinkpad laptop where all the data were stored. The tracking accuracy of the ETG was 0.5°, and its tracking range was 80° (horizontal) × 60° (vertical). A 3-point calibration method was used to calibrate the subjects’ eyes. Since visual symbols on the map (e.g., a point label indicating a building) were much smaller than objects in the real environment (e.g., a road), two calibration points were on the map (e.g., on two location labels), and one point was on the environmental object (e.g., on the center of the door of a building). We checked the calibration before starting each route using the following simple procedure: the experimenter first determined three labels on the map and three targets in the surrounding environment. The experimenter then required the subjects to look at the labels and targets one by one. Through the real-time video that was overlaid by the subjects’ fixations, the experimenter could know whether the subjects were looking at the labels or targets correctly. If not, we recalibrated and checked the calibration results again. However, we did not recalibrate during the route-following task to avoid disturbing the subjects.

### 3.2. Data Preprocessing

**Figure 2:**
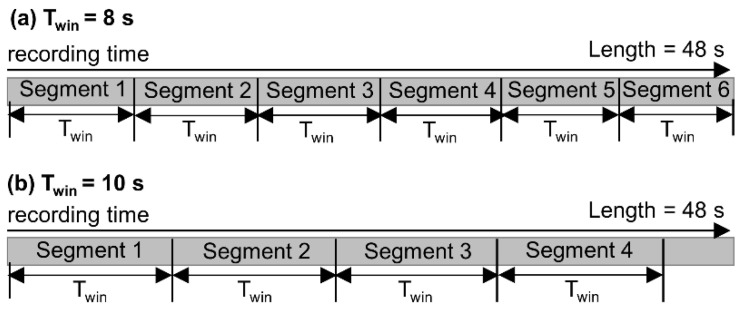
Data segmentation. (**a**) If a recording = 48 s and T_win_ = 8 s, the recording is divided into 6 segments; (**b**) for the same recording, if T_win_ = 10 s, the recording is divided into 4 segments and the last 8 s is ignored.

1. *Data quality check*. Five subjects were excluded due to calibration failure or recording failure. For the recordings of the remaining 39 subjects, we excluded recordings whose tracking ratio was below 70%, resulting in 146 total recordings (Table 1). The mean (M) tracking ratio of the recordings was 93.37%, and the standard deviation (SD) was 4.99%. Since there was no time limit for the wayfinding tasks and the subjects walked with different speeds, the durations of the recordings varied from 87.46 s to 732.53 s (M = 418.58 s, SD = 102.02 s).

2. *Fixation filtering*. We identified fixations from the raw gaze data using the SMI Event Detection algorithm, the default fixation filter of the SMI BeGaze v3.7 software [39]. The algorithm addressed head movements of subjects and thus was considered to be more capable of processing gaze data from real environments than traditional methods, such as the I-VT and I-DT algorithms [42]. The algorithm classified raw gaze data into three types of events: fixations, saccades and blinks. These data were then used for feature extraction.

3. *Data segmentation*. We divided each recording into segments of equal length (Figure 2). Hereafter, the segment length is referred to as the *time window size* (T_win_). For example, if a recording was 103 s and T_win_ = 10 s, then the recording was divided into 10 segments, with each segment being 10 s, and the last 3 s was ignored. To explore the influence of T_win_ on biometric recognition performance, we varied T_win_ from 5 s to 100 s with a step of 5 s, resulting in 20 windows (i.e., T_win_ in (5 s, 10 s, 15 s, …, 100 s)). For a given T_win_, there was no data overlap between segments. More importantly, the visual stimuli between any two segments were different because the subjects were moving. The adjacent video segments, however, might have similar scenes. For instance, a building might exist in both Segments 1 and 2. Table 2 shows the number of segments in each time window size.

### 3.3. Feature Extraction

For each data segment, we extracted the following five sets of features.

**Figure 3:**
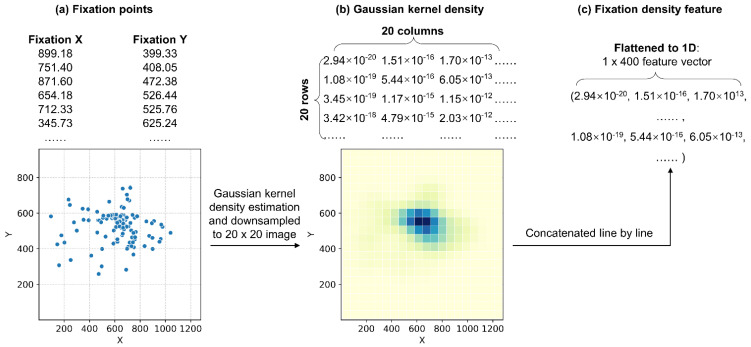
Calculation of fixation density features.

**Figure 4:**
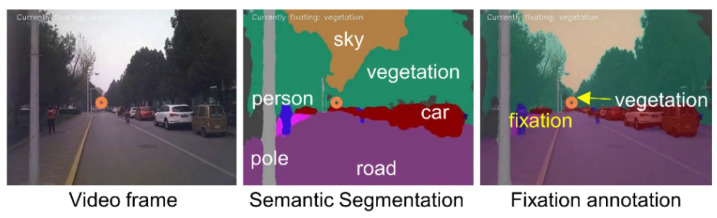
Semantic segmentation of the videos and fixation annotation.

1. *Basic statistical features*. As shown in Table 3, we extracted 11 eye movement metrics based on fixation, saccade and blink data (e.g., fixation duration, saccade amplitude, saccade acceleration and blink duration). We then computed eight statistics of these metrics: mean, standard deviation, median, max, min, 1/4 quantile, 3/4 quantile and skewness. In addition, we computed the fixation frequency, saccade frequency, blink frequency, scanpath convex hull area and scanpath length. This resulted in a total of 93 (11 × 8 + 5) features in this feature set. Many of these features have been explored in previous work (e.g., [5,23,43]) and have been proven effective for biometric recognition in laboratory environments.

2. *Pupillary response features*. Evidence has shown that pupillary responses are related to mental workload and that pupil diameter and pupil dilation are effective indicators to measure mental workload [44,45,46]. Pupil-based features have been used to recognize individuals in previous studies [25,43,47]. We computed the abovementioned eight statistics for the pupillary diameter (average of the left and right pupils) as the pupillary *response features*.

3. *Fixation density features*. Similar to Rigas and Komogortsev’s ‘fixation density maps’ [48], we computed the spatial fixation density using a Gaussian kernel (Figure 3). Note that the density was based on the two-dimensional (2D) screen *xy* coordinates of the fixations (within 1280 × 960) rather than real-world 3D coordinates. However, a 1280 × 960 vector is too large and contains redundant information. Since spatial aggregation is commonly used in analyzing spatial distributions of eye movements [49,50], we then downsampled the density to a 1D (1 × 400) vector and used it as the *fixation density* features. A 20 × 20 (=1 × 400) vector was deemed appropriate because it could show general characteristics of the spatial distribution while maintaining a sufficient level of detail.

4. *Fixation semantic features*. Since the subjects were constantly moving in a real environment, we explored whether individual subjects have unique traits when paying attention to particular objects in the environment. We first conducted semantic segmentation for the recorded videos frame by frame using Deeplabv3+ [51], which was trained using the Cityscapes dataset [52] (Figure 4). Each pixel in the video frames was assigned one of 19 object classes, such as road, car, person, person, sky and vegetation. We then overlaid the fixations on the segmented video frames and annotated the fixations with corresponding labels. An evaluation conducted by Dong et al. [53] indicated that the accuracy of this fixation annotation method was 90.7%. Note that these steps could not detect whether fixations were on the map (i.e., the subjects were reading the printed map) because the Cityscapes dataset did not contain a ‘map’ class. To address this issue, we used a scale-invariant feature transform (SIFT) algorithm [54] to detect whether video frames contained the map. We then annotated those fixations on the map with the ‘map’ label. Finally, we computed the fixation duration and fixation count for each object class (19 classes plus the ‘map’ class) as the *fixation semantic features*, resulting in a total of 40 (2 × 20) features in this feature set. Table 4 shows some examples of the fixation semantic features of Subject S01 on Route 1. Note that the fixation count (FC) and fixation duration (FD, milliseconds, ms) of only four object classes are displayed. It is seen from Table 4 that, for instance, in the first 50 s of walking in Route 1 (T_win_ = 50 s, Segment ID = 1, the first line), Subject S01 allocated 9, 4 and 23 fixations on the building, person and road, respectively. These fixations corresponded to 1365, 1531 and 5108 ms durations, respectively. However, the subject did not look at the map (FC-map = 0). The distribution and descriptive statistics of all fixation semantic features are shown in Supplementary Table S1.

Note that semantic features are likely to represent the environment’s characteristics rather than an individual’s distinctive traits. For instance, if an environment was more crowded with persons, then there was more likely a higher fixation duration/count on persons. Therefore, we adjusted the fixation semantic features using the method in Dong et al. [53]. For a given route and a subject, we averaged the total pixels of each object class across all video frames, denoted as *N*_pixel_. We then divided the original fixation duration/count by *N*_pixel_. If an environment had more persons, this adjustment decreased the fixation duration/count on persons.

In previous studies, the classifier did not learn information about the visual stimulus itself but was trained using subjects’ eye movement trajectories to the stimulus. In this study, the fixation semantic features provided information on both the stimuli (environment) and the eye movements to the classifier.

5. *Saccade encoding features*. This feature set was proposed by Bulling et al. [55]. We chose this feature set because it was designed for intention prediction in the real world. Bulling et al. demonstrated that it was effective to distinguish office activities such as browsing the internet, watching a video and copying text. We intended to explore whether it was also effective for recognizing user identities in a real environment. In this method, the saccades were first encoded into a string of characters based on their directions (4- or 8-cardinal directions) and amplitudes. The string was then scanned using a sliding window, and the substrings within the sliding window were called ‘micropatterns’. By varying the window length and moving the window forward, different micropatterns were produced and counted as features. Please refer to Bulling et al. [55] for feature description details. There were 40 features in this feature set.

### 3.4. Classification and Cross-Validation

**Figure 5:**
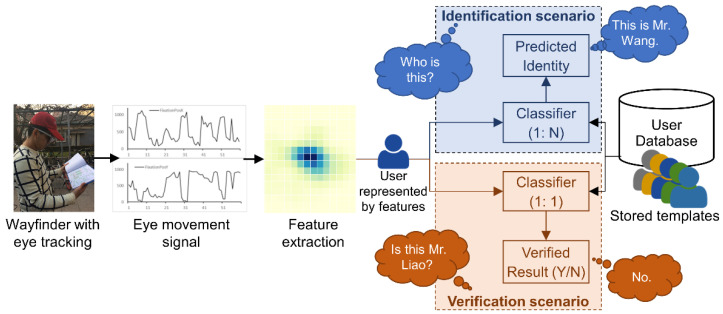
The framework of user identification and verification.

According to Jain et al. [2], a biometric recognition system can be operated in two scenarios: *identification* mode and *verification* (or *authentication*) mode (Figure 5). The identification scenario refers to matching a given user (represented by features) to all the stored templates (candidates) in the database (one-to-many match) and finding a best-matched candidate as the predicted identity, whereas the verification scenario refers to determining whether a claimed identity matches the corresponding template in the database or not (one-to-one match). The verification scenario requires the user to claim an identity. In practical applications, the biometric recognition problem is usually transferred to classification problems: the identification scenario is transferred to a multiclass classification problem, whereas the verification scenario is considered a binary (0 or 1) classification problem. Therefore, the identification and verification scenarios can be realized using machine learning methods (e.g., SVM, KNN and random forest).

We used a random forest to perform the classification because a random forest exhibited better performance in our pilot study. As an ensemble method, a random forest randomly selects subsets of the data to grow decision trees and make final predictions based on majority votes [56]. The randomness makes a random forest resilient to overfitting of the data [57]. A random forest was also adopted in previous work, such as [58,59]. We implemented the above classification and cross-validation process using the Scikit-Learn Python library [60], a commonly used machine learning open-source library. Two key parameters of the random forest, the number of trees (*n_estimators*) and the maximum number of features (*max_features*) for each tree, were set to 500 and *sqrt* (*n_features*), respectively. These optimal values were determined using randomized search cross-validation.

Classification and cross-validation were conducted for both the identification and verification scenarios.

**Figure 6:**
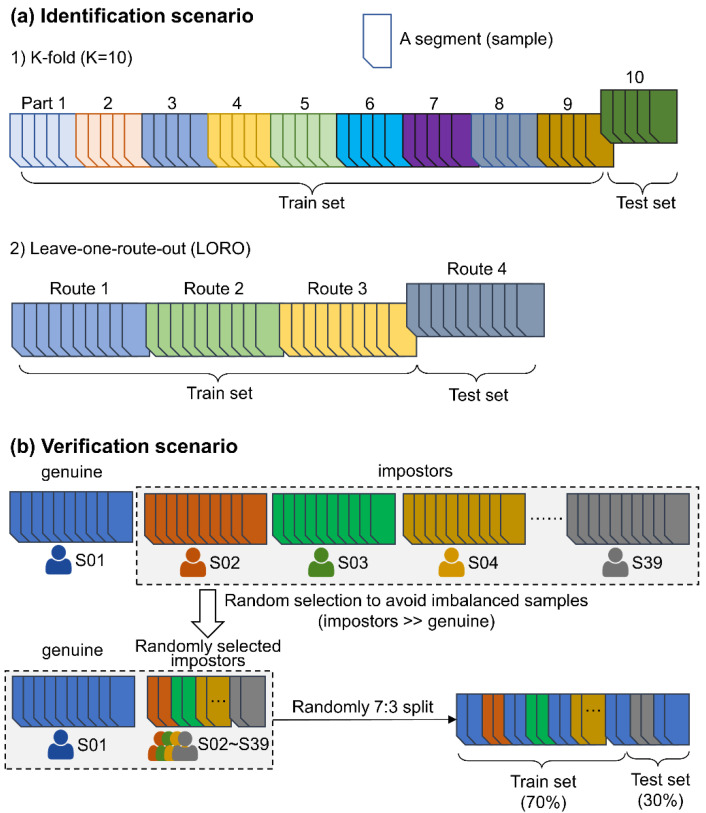
Classification and cross-validation of the (**a**) identification and (**b**) verification scenarios. Each segment was associated with a person’s identity and was considered an independent sample in the classification.

- *Identification scenario.* We used two cross-validation methods: a K-fold (K = 10) and a leave-one-route-out (LORO) method (Figure 6a). In the 10-fold classification, we pooled all data of the four routes and randomly split the data into 10 parts. In a round of classification, we used nine parts (e.g., Parts 1~9) of the data to train the classifier and then used the remaining part (e.g., Part 10) to test. This process was repeated until all 10 parts were tested. Unlike the K-fold classification, the LORO method first used three out of the four routes (e.g., Routes 1~3) to train the classifier and then used the remaining route (e.g., Route 4) for testing. This process was repeated until all four routes were tested. The LORO method rigorously ensured that the stimuli between the training and test data were completely different. We used the LORO method to test the generalizability of the classifier to identify subjects in new environments. In the identification scenario, for each combination of a classification method (10 rounds in 10-fold and four routes in LORO), a time window size (20 windows) and a feature set (5 feature sets plus combining all feature sets, hereafter referred to as *combined features*), we conducted a classification run, resulting in a total of 1800 (15 × 20 × 6) runs.
- *Verification scenario.* This scenario was carried out with binary classification. In a round of classification (Figure 6b), the data of a given subject acted as positive (genuine) samples (e.g., S01), and the other subjects acted as negative (impostor) samples. This was repeated until all subjects acted as genuine samples once. To avoid an imbalanced number of samples between genuine and impostor samples (impostors ≫ genuine), we randomly selected impostors to maintain balance. In a round of classification, the training and testing sets were split at a ratio of 7:3. In the verification scenario, we conducted a classification run for each combination of a subject (39 subjects), a time window size and a feature set, resulting in a total of 4680 (39 × 20 × 6) runs.

### 3.5. Evaluation Metrics

We used the following metrics to evaluate the results.

- *Accuracy* and *rank-k identification rate.* In the identification scenario, for a given segment (user), the classifier computes the match probability of the given segment to each candidate in the database. The biometric system then ranks the candidates based on their probabilities. There are two strategies for the system to make decisions. (1) The system uses the rank-1 (the most likely) candidate as the final predicted user. If the predicted user is true, we then consider that the system correctly identifies the segment. For all tested segments, *accuracy* or *rank-1 identification rate* (Rank-1 IR) is defined as the number of correctly identified segments divided by the total number of tested segments. (2) The system can use top-k candidates (i.e., the most likely k candidates) to predict the given segment. If the top-k candidates contain the true user, we still consider that the system correctly identifies the given segment within rank-k candidates. In other words, the system can give k tries to identify the given segment. Obviously, with the increase in k, it is easier to make a correct identification. For all tested segments, the *rank-k identification rate* (Rank-k IR) is the number of correctly identified segments within the top-k candidates divided by the total number of tested segments. Therefore, the accuracy, or Rank-1 IR, is a special case of Rank-k IR. The definition of the identification accuracy can be easily transferred to the verification scenario. The *verification accuracy* is defined as the number of correctly verified segments divided by the total number of tested segments.
- *Receiver operating characteristic* (ROC) curve. The ROC curve is plotted as the true positive rate (TPR) on the y-axis versus the false positive rate (FPR) on the x-axis [61]. The equal error rate (EER) is the point where the TPR equals the FPR, indicating the possibility that a classifier misclassifies a positive segment as a negative segment or vice versa.
- *Cumulative match characteristic* (CMC) curve. The CMC curve is only applicable to the identification scenario. The CMC curve plots the identification rate on the y-axis for each rank (i.e., k varies from 1 to 39) on the x-axis.

## 4. Results

### 4.1. Identification Scenario: 10-Fold Classification

**Figure 7:**
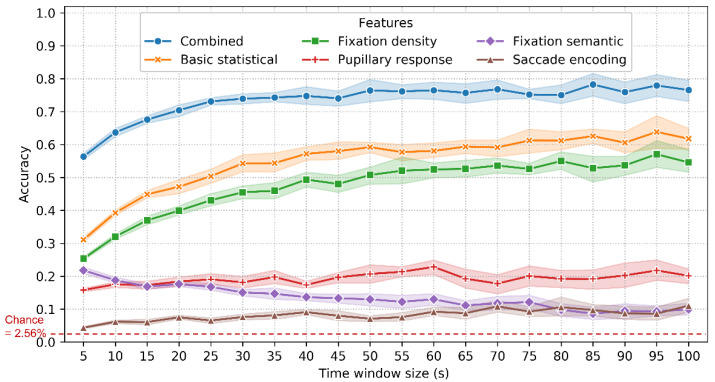
Accuracy and 95% confidence interval (CI) with varying time window size and feature types using the 10-fold classification. Refer to Table A1 in Appendix A for detailed accuracy values.

**Figure 8:**
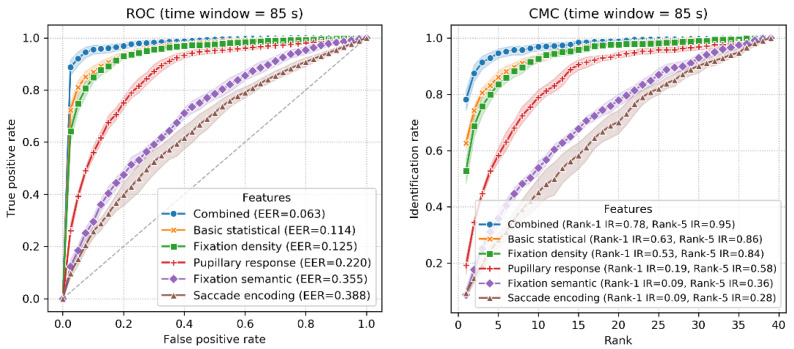
ROC (**left**) and CMC (**right**) curves in the 10-fold classification (T_win_ = 85 s).

The accuracy of identification using 10-fold classification is shown in Figure 7. The ROC and CMC curves when T_win_ = 85 s are shown in Figure 8. The combined features achieved an accuracy between 56% (T_win_ = 5 s, SD = 1.5%) and 77% (T_win_ = 100 s, SD = 5.5%), with a chance level of 1/39 (2.56%). The highest accuracy was 78% (T_win_ = 85 s, 95 s; SD = 6.1%, 5.5%). When T_win_ = 85 s, the best EER achieved was 6.3%. These results are quite promising, given that this involves implicit and stimulus-independent identification of real-world activities.

The accuracy of the combined features is significantly higher than that of the basic statistical features (31~64%; SD = 1.2~8.5%), fixation density features (25~57%; SD = 0.9~6.6%), pupillary response features (16~22%; SD = 0.9~6.6%), fixation semantic features (9~22%; SD = 1.2~3.7%) and saccade encoding features (4~11%; SD = 0.5~5.1%).

For the combined, basic statistical and fixation density features, the accuracy increases as the time window size increases from 5 s to 40 s and then remains relatively stable for subsequent window sizes. In contrast, the accuracy of the fixation semantic features decreased from 22% (SD = 1.6%) to 10% (SD = 2.9%) when the time window increased from 5 s to 100 s. This is probably because, with the increasing time window size, this feature set is more likely to represent the characteristics of the environment rather than the distinctive traits of the individuals. The accuracy of the pupillary response and saccade encoding features was insensitive to the changes in time window size.

### 4.2. Identification Scenario: LORO Classification

**Figure 9:**
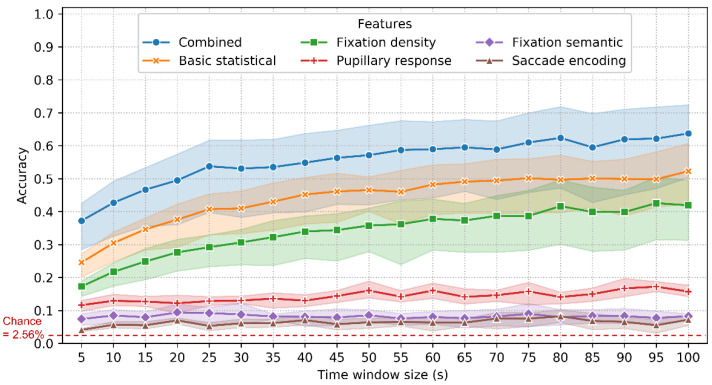
Accuracy and 95% CI with varying time window size and feature types using the LORO classification. Refer to Table A2 in Appendix A for detailed accuracy values.

The accuracy when using the LORO method is shown in Figure 9. Since the classifier learned no information about the testing environment in this method, it is unsurprising that the accuracy when using the LORO method for identification is lower than that when using the 10-fold classification (Figure 7). However, the relative performance between the six feature sets was the same as in the 10-fold classification. The combined features achieved an accuracy between 37% (T_win_ = 5 s, SD = 8.9%) and 64% (T_win_ = 100 s, SD = 13.0%), followed by the basic statistical (25~52%; SD = 4.4~11.4%), fixation density (17~43%; SD = 3.0~12.1%), pupillary response (12~17%; SD = 1.4~4.0%), fixation semantic (7~9%; SD = 2.3~4.0%) and saccade encoding features (4~8%; SD = 0.5~3.2%).

**Figure 10:**
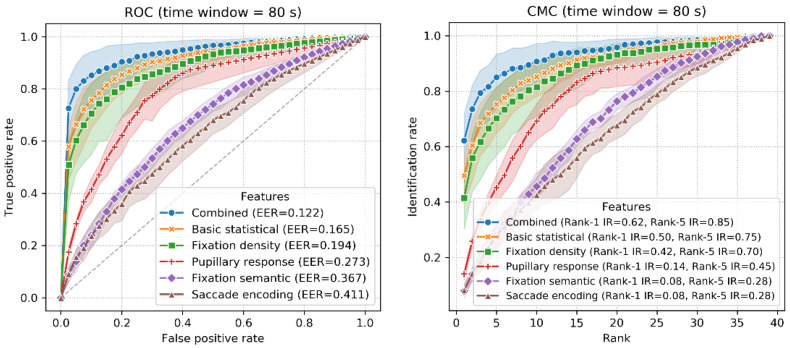
ROC (**left**) and CMC (**right**) curves using the LORO classification (T_win_ = 80 s).

The ROC and CMC curves when T_win_ = 80 s are shown in Figure 10. When T_win_ = 80 s, the best EER of 12.2% was exhibited by the combined features with Rank-1 IR = 62%, followed by the basic statistical (EER = 16.5%, Rank-1 IR = 50%), fixation density (EER = 19.4%, Rank-1 IR = 42%), pupillary response (EER = 27.3%, Rank-1 IR = 14%), fixation semantic (EER = 36.7%, Rank-1 IR = 8%) and saccade encoding features (EER = 41.1%, Rank-1 IR = 8%).

The best accuracy of 64% (EER = 12.1%) achieved by the combined features was moderate, considering that the identification was performed in completely new environments. The LORO method ensured that the classifier had learned no information about the test environments.

### 4.3. Verification Scenario

**Figure 11:**
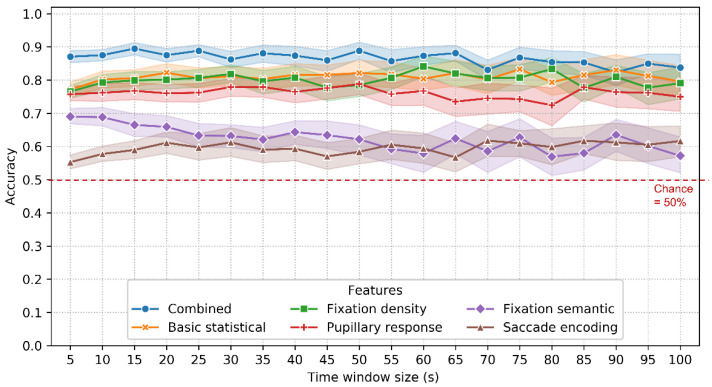
Accuracy and 95% CI with varying time window size and feature types in the verification scenario. Refer to Table A3 in Appendix A for detailed accuracy values.

**Figure 12:**
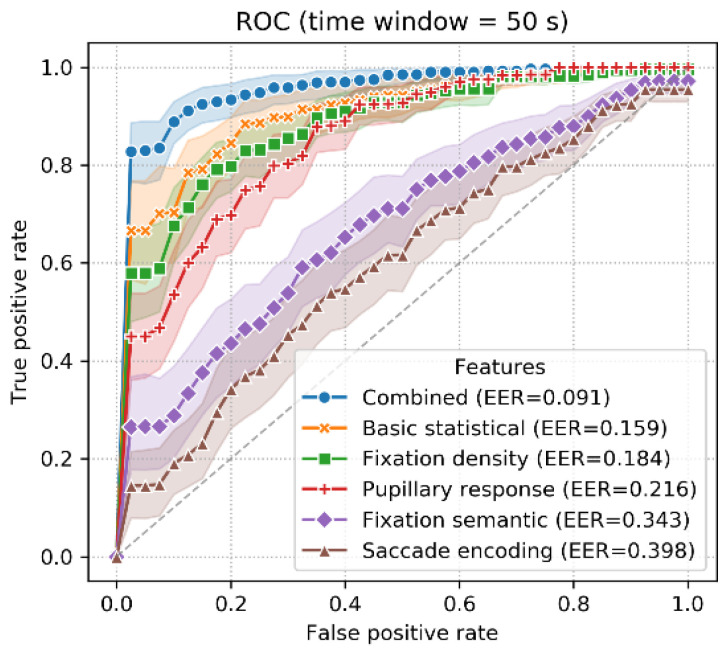
ROC curve for the verification scenario (T_win_ = 50 s).

As shown in Figure 11, in the verification scenario, the highest accuracy was achieved when using the combined features (82~89%; SD = 5.2~13.4%), followed by the basic statistical (77~83%; SD = 7.0~15.5%), fixation density (77~84%; SD = 6.8~15.3%), pupillary response (74~77%; SD = 7.1~19.1%), fixation semantic (57~69%; SD = 7.7~17.9%) and saccade encoding features (55~62%; SD = 6.4~17.1%). The ROC curve for T_win_ = 50 s is shown in Figure 12. The best EER of 9.1% was achieved using the combined features, and the worst EER of 39.8% was achieved using the saccade encoding features.

## 5. Discussion

### 5.1. Performance, Feature Importance and Time Window Size

In this study, we tested 182 features (the 1 × 400 fixation density vector was considered as one feature), which were grouped into five feature sets. The general importance of the feature sets (basic statistical > fixation density > pupillary response > fixation semantic > saccade encoding) is in line with previous studies. Combining the five feature sets could lead to the best accuracy of 78% (EER = 6.3%) and 89% (EER = 9.1%) in the identification and verification scenarios, respectively. These promising results are comparable to those of many studies with lab environments, such as Liang et al. [62] (accuracy: 82%), Rigas et al. [26] (accuracy: 70.2%), Saeed [6] (accuracy: 85.72%) and Schröder et al. [19] (accuracy: 86.7%). Note that it may be unfair to compare the performances of different studies because they vary in a series of aspects, such as stimuli, tasks, number of subjects, eye movement features, classification methods and evaluation metrics.

It is worth noting that these results were achieved using eye movement data that were captured at a relatively lower sampling frequency (60 Hz). As mentioned in Section 2.2, such a frequency is much lower than Holland and Komogortsev’s [23] recommendation of 250 Hz and may not be able to fully characterize subjects’ saccade dynamics. As a result, the low frequency might have affected the contribution of different types of features (especially those saccade-related features) in the classification. For instance, the saccade encoding features exhibited the lowest performance in both the identification and verification scenarios (Figure 7, Figure 9 and Figure 11). However, our results provide evidence of the feasibility of implementing eye movement biometric systems using a relatively lower frequency. This is important for developing mobile and wearable biometric systems in real environments.

The fixation density features (1D 1 × 400 vector) reached an accuracy of 25~57% in the identification scenario (Figure 7) and 77~84% in the verification scenario (Figure 11). This confirms Rigas and Komogortsev’s [48] finding that the spatial distribution of eye movements with dynamic visual stimuli was able to distinguish individuals. Note that in Rigas and Komogortsev’s experiment, their visual stimuli were dynamic but the subjects were static. In our experiment, both the visual stimuli (i.e., the real environment) and the subjects were dynamic. Our results indicate that fixation density features are robust to changes in the environment and the subjects.

The pupillary response features (N = 8) achieved an accuracy of 16~22% in 10-fold and 12~17% in LORO identification, which is comparable to Liao et al. [29]’s accuracy of 12~18% (32 subjects, task: reading maps). However, the accuracy is lower than Bednarik et al.’s [25] best accuracy of 50~60% (12 subjects, tasks: reading text, tracking a moving cross and watching a static image). Our accuracy is also lower than Darwish and Pasquier’s [47] results (precision: 71.1~85.8%; 22 subjects; and tasks: viewing images and connecting dots). The lower accuracy of this study may partly be because the pupil responses are easily affected by the light condition changes in the real environment.

This study first used fixation semantic features (N = 40) for biometric recognition. This feature set contains characteristics of both the subjects’ visual behavior and the visual stimuli. However, its low accuracy (≈10% in identification and ≈60% in verification; Figure 7, Figure 9 and Figure 11) indicates that what people see in the environment is an indistinctive feature to recognize people. In addition, with a longer time length, the fixation semantic features become representative characteristics of the visual stimuli rather than the subjects. Therefore, the accuracy decreases with increasing time length in the 10-fold classification (Figure 7).

We tested the influence of the time window size on the accuracy of both the identification and verification scenarios. In the identification scenario of 10-fold classification (Figure 7), the accuracy increased (56~75%) in the first 40 s and then remained relatively stable (75~78%) in the last 60 s. An interesting finding is that in the verification scenario, the accuracy was insensitive to the changes in time window size regardless of the features used (Figure 11), which is different from the identification scenario (Figure 7). This means that a few seconds of eye movement data (e.g., 5 s) is sufficient for verification in real-world wayfinding. This is of great importance for continuous verification in real-world activities. These time lengths (40 s and 5 s) were shorter than Schröder et al. [19]’s 90 s of using TEX and RAN datasets.

### 5.2. Reliability of Eye Movement Features

One important issue of gaze-based applications is to measure the reliability of eye movement features [63]. For example, in a study investigating the sensitivity of eye movement metrics to changes in cognitive load, Marandi et al. [64] found that saccadic velocity amplitude, saccade peak velocity, saccade duration and fixation duration could provide good test-retest reliability. Other metrics, such as smooth pursuit, saccadic latency, saccade left-right asymmetries and fixation stability, were proven to be reliable [65,66,67]. Many of these metrics were included in our basic statistical features, and the basic statistical features exhibited better performance than other types of features in both the identification (31~64% in 10-fold and 25~52% in LORO classification) and verification (77~83%) scenarios. The reliability of eye movement features is affected by more factors in real-world activities (e.g., changing traffic, weather and light conditions, moving vehicles and persons, and the low sampling rate of wearable eye trackers, as mentioned in Section 2.2) than in the laboratory. However, in this study, we did not test the reliability of the features that were extracted from eye movement data in real environments, which is a major limitation of this study.

### 5.3. Task-Dependence of Eye Movements and Multilevel Tasks of Wayfinding

Another important issue is that wayfinding contains multilevel tasks (subactivities) such as map reading, object search, walking, route confirmation and reorientation [68]. In real-world wayfinding, it is difficult to distinguish these sub-activities accurately because of their dynamic nature [21]. For instance, a wayfinder may read a map to reorient him/herself while walking. Furthermore, previous studies have demonstrated that eye movements are task-dependent [12,55,69,70,71]. For example, early in 1967, Yarbus [71] found that different picture viewing tasks (e.g., memorizing the picture and guessing the wealth of the people in the picture) could lead to different eye movement patterns. Bulling et al. [56] showed that eye movement signals were associated with five office activities such as copying a text, reading a paper and browsing the Web. In a real-world wayfinding experiment, Liao et al. [12] predicted five wayfinding tasks from subjects’ eye movement data and found that some basic statistical features, such as fixation dispersion, fixation duration, fixation frequency, saccade latency, saccade duration and saccade frequency, were significantly influenced by navigation tasks (i.e., they were task-dependent features). Similarly, some features such as fixation semantics are stimulus-dependent. In the present study, different segments may be associated with different subactivities, and thus the classification may be performed across both stimuli and subactivities. In the future, it is important to investigate whether and how the task-dependence and stimulus-dependence of eye movement features affects biometric recognition performance.

### 5.4. Limitations

There are two limitations in this study that can drive future research.

First, the present study is limited by the small sample size (39 subjects × 4 routes) of the wayfinding experiments. This is mainly due to the difficulties in conducting real-world eye tracking experiments [54] (as mentioned in Section 2.2). More data (more routes, more subjects and more tasks) need to be collected to validate the current results.

Second, only wayfinding tasks were tested in this study. It is unknown whether eye movement biometrics can be realized for other types of real-world activities. In addition, when there are multiple types of activities, it is important to test whether eye movement biometric recognition can be performed across activities (i.e., task-independent).

## 6. Conclusions

Wearable eye tracking was used in this work to accomplish biometric recognition in real-world pedestrian wayfinding. We investigated the performance of five feature sets and 20 temporal window sizes in both biometric identification and verification scenarios. In the identification and verification situations, the best accuracies of 78% (EER = 6.3%) and 89% (EER = 9.1%) were attained by merging all the feature sets. The combined features outperformed the basic statistics, fixation density, pupillary response, fixation semantic, and saccade encoding features in both scenarios. When the time window size was extended in the identification scenario, the accuracy first increased in the first 40 s and then remained relatively stable in the following window sizes. In the verification scenario, however, the accuracy was unaffected by the size of the time window, indicating that a few seconds of eye movements (e.g., 5 s) was sufficient for biometric verification. The findings showed that employing wearable eye tracking to accomplish biometric recognition in real-world pedestrian navigation is possible.

## Figures and Tables

**Table 1:** Mean durations and number of recordings in each route.

Route	Mean Duration (s)	SD	Number of Recordings
Route 1	361.48	48.79	38
Route 2	396.21	59.51	37
Route 3	527.33	121.75	38
Route 4	384.19	59.77	33
Overall	418.58	102.02	146

**Table 2:** Number of segments in each time window size.

T_win_ (s)	5	10	15	20	25	30	35	40	45	50	55	60	65	70	75	80	85	90	95	100
Segment count	11974	6101	4110	3102	2503	2098	1793	1570	1403	1270	1166	1064	992	923	867	822	772	736	694	670

**Table 3:** Basic statistical features.

Eye Movement Metric		Statistic	N
Fixation	Fixation duration, fixation dispersion	mean, standard deviation, median, max, min, 1/4 quantile, 3/4 quantile and skewness	16
Saccade	saccade duration, saccade amplitude, saccade velocity, saccade latency, saccade acceleration, saccade acceleration peak, saccade deceleration peak and saccade velocity peak		64
Blink	blink duration		8
Fixation frequency, saccade frequency, blink frequency, scanpath convex hull area and scanpath length			5

**Table 4:** Examples of fixation semantic features (before adjustment). FC: fixation count. FD: fixation duration.

Subject ID	Route	Window Size	Segment ID	FC- Building	FC- Map	FC- Person	FC- Road	FD- Building	FD- Map	FD- Person	FD-Road
S01	Route 1	50	1	9	0	4	23	1365	0	1531	5108
S01	Route 1	50	2	3	0	0	18	815	0	0	3795
S01	Route 1	50	3	17	0	1	33	3478	0	167	8400
S01	Route 1	50	4	8	0	11	19	1448	0	2380	3212
S01	Route 1	50	5	2	0	0	31	333	0	0	6040

## Data Availability

The raw eye tracking data, including video records, cannot be publicly published due to the need to preserve the privacy of research subjects and the resulting ethical difficulties. Despite this limitation, using one subject, we created tidy sample data of five types of features encoded from raw data. The sample data can be found on figshare: https://doi.org/10.6084/m9.figshare.19443503.v1 (accessed on 6 April 2022).

## Supplementary Materials

The following supporting information can be downloaded at: https://www.mdpi.com/article/10.3390/s22082949/s1, Table S1: Descriptive statistics of fixation semantic features.

## Appendix A

**Table A1:** Mean accuracy of the 10-fold identification scenario.

Time Window Size (s)	Basic Statistical	Combined	Fixation Density	Pupillary Response	Saccade Encoding	Fixation Semantic
5	0.311	0.564	0.254	0.158	0.044	0.218
10	0.393	0.637	0.321	0.175	0.062	0.188
15	0.449	0.676	0.370	0.173	0.061	0.169
20	0.472	0.704	0.399	0.184	0.075	0.176
25	0.504	0.731	0.431	0.191	0.065	0.168
30	0.542	0.739	0.455	0.182	0.076	0.151
35	0.544	0.743	0.460	0.197	0.081	0.147
40	0.572	0.748	0.494	0.174	0.091	0.136
45	0.580	0.741	0.480	0.197	0.080	0.133
50	0.592	0.765	0.507	0.207	0.071	0.130
55	0.577	0.762	0.521	0.214	0.076	0.122
60	0.581	0.765	0.524	0.228	0.092	0.130
65	0.594	0.757	0.526	0.193	0.088	0.111
70	0.591	0.768	0.536	0.178	0.109	0.118
75	0.612	0.752	0.526	0.201	0.092	0.121
80	0.612	0.750	0.550	0.192	0.106	0.098
85	0.626	0.783	0.528	0.192	0.097	0.086
90	0.606	0.759	0.537	0.202	0.087	0.095
95	0.638	0.780	0.571	0.218	0.087	0.093
100	0.618	0.766	0.546	0.201	0.111	0.100

**Table A2:** Mean accuracy of the LORO identification scenario.

Time Window Size (s)	Basic Statistical	Combined	Fixation Density	Pupillary Response	Saccade Encoding	Fixation Semantic
5	0.246	0.372	0.173	0.116	0.041	0.074
10	0.305	0.427	0.217	0.129	0.057	0.085
15	0.346	0.467	0.249	0.127	0.055	0.080
20	0.376	0.495	0.276	0.122	0.070	0.094
25	0.407	0.538	0.292	0.128	0.053	0.092
30	0.410	0.531	0.307	0.130	0.061	0.088
35	0.430	0.535	0.323	0.136	0.062	0.082
40	0.452	0.549	0.340	0.130	0.071	0.081
45	0.461	0.563	0.344	0.144	0.058	0.079
50	0.466	0.571	0.358	0.160	0.064	0.085
55	0.460	0.587	0.362	0.142	0.065	0.076
60	0.482	0.590	0.378	0.160	0.064	0.080
65	0.491	0.595	0.373	0.141	0.063	0.077
70	0.494	0.589	0.387	0.146	0.076	0.082
75	0.502	0.610	0.387	0.158	0.075	0.090
80	0.497	0.624	0.417	0.141	0.082	0.080
85	0.501	0.595	0.399	0.150	0.068	0.083
90	0.499	0.620	0.399	0.167	0.066	0.083
95	0.499	0.622	0.426	0.172	0.055	0.078
100	0.522	0.637	0.419	0.157	0.072	0.083

**Table A3:** Mean accuracy of the verification scenario.

Time Window Size (s)	Basic Statistical	Combined	Fixation Density	Pupillary Response	Saccade Encoding	Fixation Semantic
5	0.772	0.870	0.765	0.757	0.553	0.690
10	0.802	0.875	0.793	0.762	0.577	0.688
15	0.806	0.895	0.799	0.768	0.590	0.665
20	0.822	0.875	0.801	0.760	0.611	0.659
25	0.806	0.889	0.807	0.762	0.597	0.633
30	0.812	0.862	0.818	0.779	0.612	0.632
35	0.803	0.881	0.796	0.779	0.590	0.621
40	0.816	0.874	0.807	0.765	0.593	0.643
45	0.816	0.860	0.777	0.776	0.570	0.634
50	0.821	0.889	0.785	0.788	0.584	0.622
55	0.818	0.857	0.807	0.758	0.605	0.592
60	0.804	0.873	0.841	0.767	0.594	0.579
65	0.821	0.881	0.820	0.735	0.567	0.624
70	0.802	0.831	0.807	0.745	0.618	0.586
75	0.833	0.868	0.807	0.743	0.609	0.627
80	0.794	0.854	0.833	0.724	0.599	0.569
85	0.815	0.853	0.777	0.778	0.617	0.580
90	0.830	0.823	0.810	0.764	0.612	0.635
95	0.813	0.850	0.777	0.762	0.605	0.602
100	0.796	0.838	0.791	0.750	0.616	0.572
