# Supplementary material for: Exploring Eye Movement Biometrics in Real-World Activities: A Case Study of Wayfinding
Source: Sensors (Basel). 2022 Apr 12;22(8):2949. doi: 10.3390/s22082949 (PMC9030773; doi:10.3390/s22082949)
Supplement: Supplementary file 1 [file sensors-22-02949-s001.zip › sensors-1587934-supplementary.pdf]

Table S1. Descriptive statistics of fixation semantic features. FC: fixation count; FD: fixation duration (ms).

Feature	Distribution	Mean	SD	Median	Range
FC-bicycle	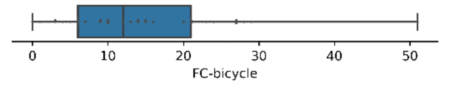	14.1	10.4	12.0	0 - 51
FC-building	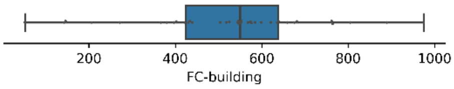	530.3	200.9	550.0	52 - 975
FC-bus	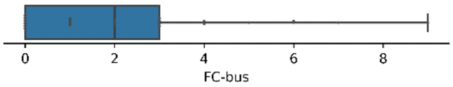	2.2	2.3	2.0	0 - 9
FC-car	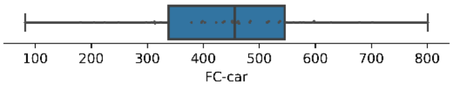	447.3	152.7	456.0	82 - 801
FC-fence	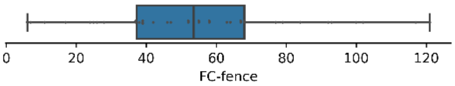	55.3	29.2	53.5	6 - 121
FC-map	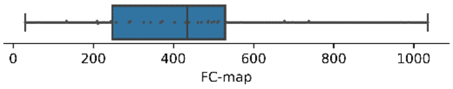	441.2	245.3	434.5	30 - 1035
FC-motorcycle	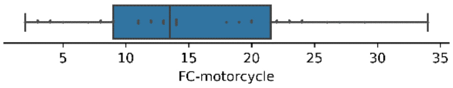	14.7	8.1	13.5	2 - 34
FC-person	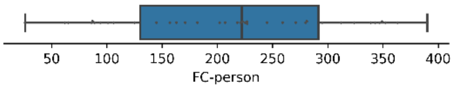	214.7	100.5	222.0	26 - 390
FC-pole	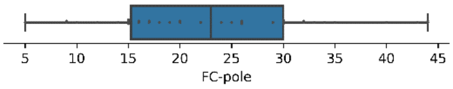	23.6	10.3	23.0	5 - 44
FC-rider	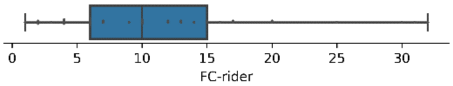	11.2	8.2	10.0	1 - 32
FC-road	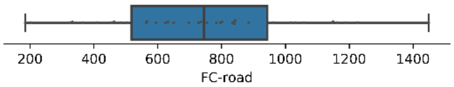	744.3	303.8	744.5	185 - 1449
FC-sidewalk	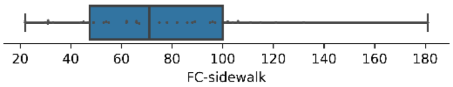	74.6	35.2	71.0	22 - 181
FC-sky	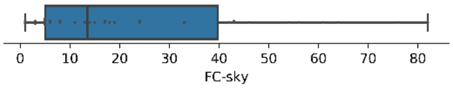	22.2	23.1	13.5	1 - 82
FC-terrain	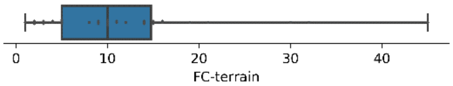	11.3	9.1	10.0	1 - 45
FC-traffic light	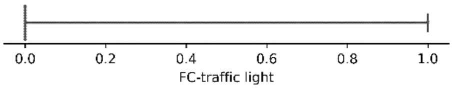	0.1	0.2	0.0	0 - 1
FC-traffic sign	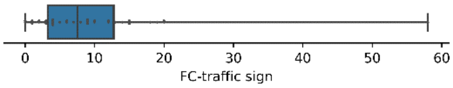	9.3	10.0	7.5	0 - 58
FC-train	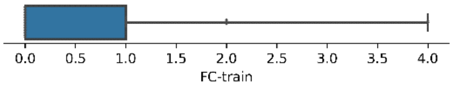	0.6	1.1	0.0	0 - 4
FC-truck	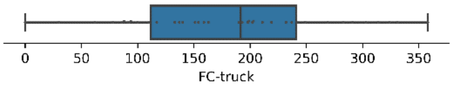	182.7	88.9	191.5	0 - 358
FC-vegetation	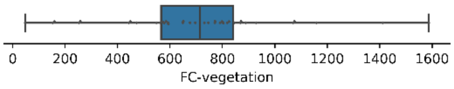	702.7	317.3	714.0	48 - 1587

FC-wall	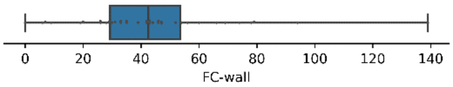	43.9	26.3	42.5	0 - 139
FD-bicycle	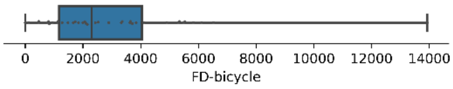	3037.9	2598.1	2292.1	0 - 13927.3
FD-building	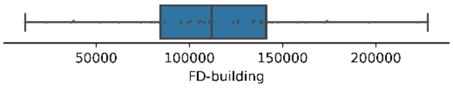	115434.5	49833.2	111808.5	11963.8 - 227850.3
FD-bus	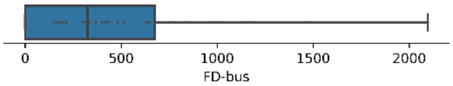	449.1	495.8	324.5	0 - 2096.6
FD-car	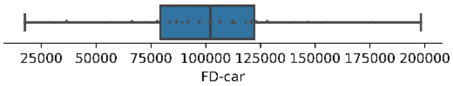	101046.3	38673.4	102145.6	17469.1 - 198245.3
FD-fence	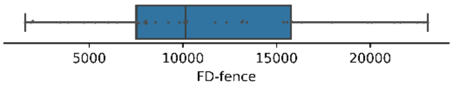	11627.5	6399.3	10142.9	1580.7 - 23076.9
FD-map	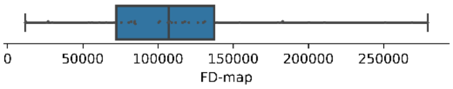	114059.0	67310.6	107061.1	11719.4 - 279410.9
FD-motorcycle	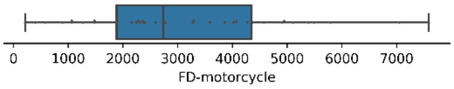	3117.6	1828.7	2737.9	216.1 - 7587.7
FD-person	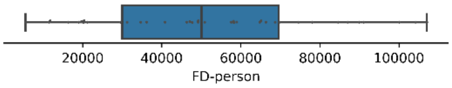	52442.0	28121.2	50030.6	5508.3 - 107038.4
FD-pole	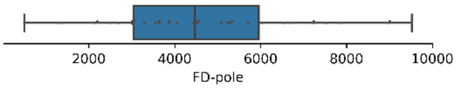	4664.6	2226.5	4467.0	499.1 - 9519.4
FD-rider	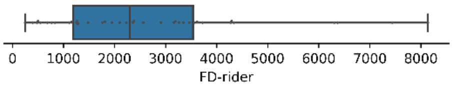	2646.9	1966.9	2296.4	249.7 - 8136.6
FD-road	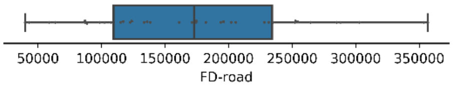	174422.2	84150.7	172764.6	39894.2 - 356756.6
FD-sidewalk	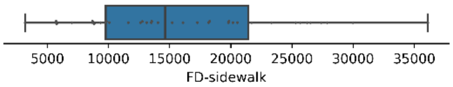	16301.9	8004.5	14632.8	3195.1 - 36109.7
FD-sky	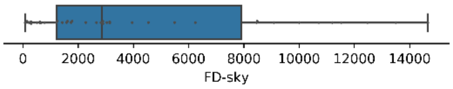	4406.6	4361.9	2861.5	83.7 - 14659.9
FD-terrain	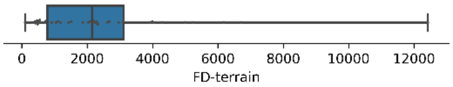	2512.1	2367.4	2146.6	103.3 - 12423.8
FD-traffic light	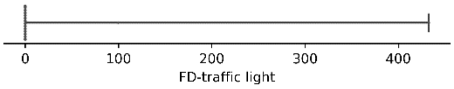	14.0	71.6	0.0	0 - 432.6
FD-traffic sign	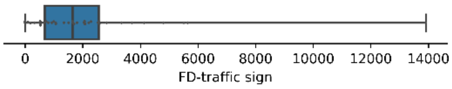	2127.0	2448.6	1647.3	0 - 13908.6
FD-train	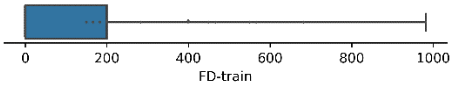	149.3	243.0	0.0	0 - 981.5
FD-truck	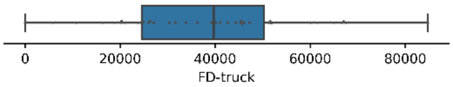	38480.9	19081.4	39698.4	0 - 84765.3
FD-vegetation	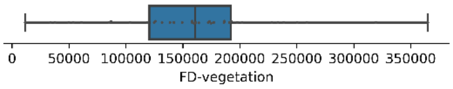	158014.4	76199.1	160650.4	11613.8 - 365008.2

FD-wall	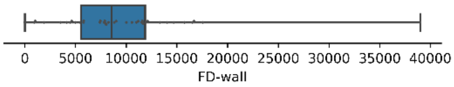	9589.9	6700.6	8528.9	0 - 38999.2
